# Cesarean Delivery in a Patient With Hypermobile Ehlers-Danlos Syndrome: A Case Report

**DOI:** 10.7759/cureus.70667

**Published:** 2024-10-01

**Authors:** Beatriz Antunes, Marta Araújo, Carlos Noversa, Joana Pedreira, Miguel Marques

**Affiliations:** 1 Anesthesiology, Unidade Local de Saúde de Braga, Braga, PRT

**Keywords:** cesarean delivery, general anesthesia, hypermobile ehlers-danlos syndrome, neuraxial anesthesia, perioperative management, regional anesthesia

## Abstract

Patients with hypermobile Ehlers-Danlos syndrome (hEDS) usually present with generalized joint hypermobility and pain, soft and hyperextensible skin with atrophic scars and easy bruising, periodontitis, mitral valve prolapse, and aortic root dilation. It may also lead to cervical spine instability or collapse of larynx cartilage/trachea, which can result in intubation difficulties and possible mucosal damage. Lung protective ventilation must be performed to prevent pneumothorax. Bruising, scoliosis, spondylosis, meningeal Tarlov cysts, increased risk for postdural puncture headache (PDPH), and resistance to local anesthetics may affect neuraxial anesthesia. This article aims to report an anesthetic approach of a 24-year-old woman with hEDS proposed for cesarean delivery. Additional past medical history included cutaneous psoriasis, polycystic ovarian syndrome, and bilateral hip dysplasia. No previous allergies were reported. She has been previously submitted to an uneventful general anesthesia. After rapid-sequence induction of general anesthesia, endotracheal intubation was performed with videolaryngoscopy. Balanced anesthesia with volatile anesthetics was used. The birth and recovery were safely managed without adverse events. hEDS can present challenges for both neuraxial anesthesia and orotracheal intubation. In this case, the initial anesthetic plan included general anesthesia and an airway approach with a videolaryngoscope. Patient positioning and padding were carefully executed to prevent bruising and joint dislocations.

## Introduction

Ehlers-Danlos syndrome (EDS) has a frequency of one in 5000 [1], with hypermobile EDS (hEDS) accounting for 90% of reported cases (classified as a rare disorder) [2]. Symptoms usually present in childhood to early adolescence, often resulting in generalized joint hypermobility associated with pain, soft and hyperextensible skin with atrophic scars and easy bruising, periodontitis, abdominal hernias, pelvic organ prolapse, marfanoid body habitus, mitral valve prolapse, and aortic root dilatation (typically of a mild degree with no increased risk of cardiac complications) [3]. Other common features include chronic fatigue, functional bowel disorders, dysautonomia, swallowing and phonation disorders, gastroesophageal reflux, obstructive sleep apnea, migraine, peripheral neuropathies, mast cell activation disorders, anxiety disorders, urogynecologic disorders, and bleeding complications [1]. The gastrointestinal tract, gravid uterus, and, less commonly, lungs, spleen, and liver are prone to spontaneous rupture [4].

Perioperative management in patients with hEDS includes assessment of patient history, namely, the specific subtype, bleeding anamnesis, EDS complications, and airway difficulties. Efforts should be made to avoid tourniquets (risk of hematoma and compartment syndrome), perform adequate patient positioning with padding (reduction of shear forces and external tissue pressure), and ensure early mobilization. In the case of positive bleeding history, desmopressin might be used and crossmatching adequate amounts of red blood cells (RBCs) must be performed. Non-invasive monitoring should be performed, keeping in mind the risk of hematoma formation by repetitive non-invasive blood pressure measurements. Postural orthostatic tachycardia syndrome is common in patients with hEDS and preoperative infusion of crystalloid and early use of vasopressors might be helpful.

Regarding airway management, care is required with mask ventilation to avoid temporomandibular joint luxation. Repeated intubation attempts can lead to bleeding and cuff pressure should be monitored closely and kept as low as possible to minimize mucosal damage, preferably using smaller endotracheal tubes. Airway pressure should be minimized due to the risk of pneumothorax. Patients with temporomandibular dysfunction, premature spondylosis, or occipitoatlantoaxial instability have an increased risk of difficult airway management.

General anesthesia can be performed as balanced anesthesia with volatile anesthetics, nitrous oxide, or as total intravenous anesthesia (TIVA). Neuromuscular relaxants are safe and monitoring of neuromuscular blockade is advised before emergence from the anesthesia.

Regarding regional anesthesia, a greater rate of postdural puncture headache (PDPH) after neuraxial blockade might be expected, due to tissue fragility. Difficulties in the technique might be encountered in the presence of spinal pathology (scoliosis, kyphosis, spinal stenosis, etc.). Isolated or multiple Tarlov cysts (cerebrospinal-fluid filled perineurial cysts) as a sign of meningeal involvement is a feature of hEDS, most of which are located in the S1 to S4 region of the spinal cord. Pre-interventional ultrasound imaging (or use of MRI) might help to rule out relevant spine pathology. In addition, local anesthetic resistance has been reported [5,6].

There is no general recommendation for the mode of delivery in specific EDS types [4]. Neuraxial regional anesthesia should be avoided in vascular EDS. Using patient-controlled IV remifentanil for vaginal delivery might be a pragmatic approach in patients with a high risk for neuraxial complications.

## Case presentation

A 24-year-old woman, ASA 2, presented for cesarean delivery due to a breech presentation. Past medical history included hEDS, cutaneous psoriasis, polycystic ovarian syndrome, and bilateral hip dysplasia. Symptoms of hEDS included joint hypermobility and pain, scoliosis, and striae. She was medicated with oral supplements (folic acid, vitamin B12, vitamin B6, vitamin D, omega 3, and essential minerals, like iron, magnesium, iodine, and ginger). No previous allergies were reported. She has been previously submitted to an uneventful general anesthesia for rhinoplasty. The preoperative analytical study revealed a hemoglobin of 11.9 g/dl, a platelet count of 306,000/mm^2^, and a coagulation study without changes. Typing and crossmatching of two RBC units was performed.

After entering the operating room, the patient was monitored according to the ASA standard. Vital signs were as follows: blood pressure of 123/87 mmHg, heart rate of 99 beats per minute (bpm) with normal sinus rhythm, and peripheral oxygen saturation (SpO_2_) of 96% in room air. Anesthetic depth was monitored with BIS® and neuromuscular blockade with TOF®. Fluid therapy with Ringer's lactate and active warming maneuvers with a thermal air blanket was started. Pre-oxygenation was initiated with a face mask and a rapid sequence induction of general anesthesia was carried out, using 200 mg of propofol and 100 mg of rocuronium. Orotracheal intubation was performed with the McGrath videolaryngoscope, and a simple 7.0 orotracheal tube was introduced. Cuff pressure was checked and general anesthesia was maintained using a mixture of air, oxygen, nitrous oxide, and sevoflurane. Invasive blood pressure monitoring was started and a bladder catheter was introduced. The patient was mobilized with great caution and the pressure areas were protected with padding. After birth, we administered 100 mcg of fentanyl. 

We opted for multimodal postoperative analgesia with 1000 mg of paracetamol, 75 mg of diclofenac, and 200 mg of tramadol. Postoperative nausea and vomiting prophylaxis was performed using 8 mg of dexamethasone. During the operation, vital signs were stable with a systolic blood pressure of 103-140 mm Hg, heart rate of 93-120 bpm without arrhythmia, and SpO_2_ of 99-100%. At the end of the surgery, 400 mg of sugammadex was administered and emergence from anesthesia and extubation were performed after obtaining a TOF ratio > 0.9 and the patient was awake (eye-opening and responding to commands). We completed the anesthesia procedure in 55 minutes (surgery lasted 32 minutes), with a total intraoperative fluid infusion volume of 600 mL, an estimated blood loss of 400 mL, and a total urinary output of 250 mL. The birth and recovery were safely managed without adverse events. In the postanesthesia care unit, a total of 8 mg of morphine and 25 mg of pethidine were administered.

## Discussion

This case illustrates a 24-year-old woman with hEDS who underwent a cesarean section under general anesthesia. 

hEDS is the most common type of EDS and is characterized by general features of EDS with less severe manifestations (e.g., mild skin stretch and scarring) [7]. The prevalence of this syndrome is approximately one in 3200. No single pathogenic gene variant has been found to be related to the cause and inheritance and its pathophysiology is not understood: unlike the other types of EDS, no structural abnormality in collagen or related proteins has yet been identified. However, it is considered to have dominant inheritance with highly variable penetrance of signs and symptoms.

The major clinical feature of hEDS includes joint hypermobility. Other common symptoms include the following (Table 1):

**Table 1:** Common symptoms often associated with hypermobile Ehlers-Danlos syndrome

Type of symptoms/disorder	Features
Musculoskeletal symptoms	Generalized joint hypermobility associated with pain [3]
Connective tissue abnormalities	Soft and hyperextensible skin with atrophic scars and easy bruising, periodontitis, abdominal hernias and marfanoid body habitus [3]; The gastrointestinal tract and gravid uterus, and, less commonly, lungs, spleen and liver, are prone to spontaneous rupture [4]
Autonomic dysfunction	Orthostatic intolerance and postural tachycardia syndrome [7,8]
Psychiatric disorders	Anxiety and depression [7,9]
Neurologic disorders	Spinal instability, migraine/tension-type headache and peripheral neuropathies [1,7]
Gastrointestinal disorders	Gastroesophageal reflux, bowel dysmotility, irritable bowel syndrome and rectal prolapse [1,7]
Genitourinary disorders	Pelvic organ prolapse and functional bladder disorder (eg, interstitial cystitis/bladder pain syndrome) [1,7]
Cardiovascular disorders	Mitral valve prolapse and aortic root dilatation (typically of a mild degree with no increased risk of cardiac complications) [3]
Other characteristics	Chronic fatigue, swallowing and phonation disorders, obstructive sleep apnea, mast cell activation disorders and bleeding complications [1]

During pregnancy, these patients might experience higher rates of complications such as increased ligament laxity, injuries from malposition in labor, abdominal wall herniation, pelvic floor weakness, rapid progression of labor, wound healing deficiency, resistance to local anesthetics, fatigue, postural tachycardia syndrome, gastroesophageal reflux, and peripheral venous insufficiency [7]. Despite these potential complications, pregnancy and delivery are relatively safe in patients with hEDS compared to patients with other forms of EDS [10]. The mode of delivery remains controversial: uterine or bowel rupture, extensive perineal trauma, and delayed wound healing are complications for both vaginal delivery and cesarean section [11]. In addition, episiotomy is related to pelvic prolapse in hEDS patients. Therefore, cesarean section may be considered as the first delivery option in this EDS subtype.

Endotracheal intubation can be challenging due to the collapse of the tracheal cartilage, cervical spine instability, and temporomandibular joint dysfunction [10]. BURP maneuver can occlude the trachea and affect the view during laryngoscopy. These difficulties might be overcome with the use of videolaryngoscopy, intubating laryngeal mask airway devices, or fiberoptic bronchoscopes. Mechanical ventilation places these patients at higher risk of oral and laryngeal hematomas and pneumothoraces. 

Neuraxial anesthesia is also related to complications such as an increased rate of PDPH. Spinal pathology might affect the technique, making previous imaging exams useful [4].

There is no definite recommendation for either general or regional anesthesia, and several case reports and case series of neuraxial anesthesia and peripheral nerve blocks have reported no complications. However, due to the possible disadvantages of neuraxial anesthesia (greater rate of PDPH, possible difficulties in the technique in the presence of spinal pathology, existence of isolated or multiple Tarlov cysts, and local anesthetic resistance) [4,5], general anesthesia was carried out in this case. It can be performed as balanced anesthesia with volatile anesthetics, nitrous oxide, or TIVA [4,11]. As mentioned above, there is an increased risk of a difficult airway, and several measures should be taken to avoid complications: careful mask ventilation (avoiding temporomandibular joint luxation), use of a smaller endotracheal tube (avoiding mucosal damage) with regular verification of the cuff pressure, and lung-protective ventilation (avoiding pneumothorax) [11,12]. We followed these guidelines, along with orotracheal intubation using a videolaryngoscope (minimizing temporomandibular and occipitoatlantoaxial joint luxation). Due to the possibility of formation of hematomas, joint dislocations, bruises on the skin, and peripheral neuropathy, mobilization and positioning were carried out with great caution, with padding and protection of pressure areas [13]. General anesthesia was maintained using a mixture of air, oxygen, nitrous oxide, and sevoflurane. The literature advocates non-invasive monitoring whenever possible. However, given the fact that some patients develop extensive hematoma from repetitive non-invasive blood pressure measurements and the lack of an EDS subtype with vascular fragility (hence decreasing the risk of vascular wall dissection with arterial cannulation), invasive blood pressure monitoring was initiated.

Postoperative care should focus on the development of bleeding and hematoma at the surgical site, as postpartum hemorrhage and complicated perineal lacerations occur more often than in the general population [14]. Early mobilization is important in order to prevent excessive deconditioning and unexpected deterioration of the musculoskeletal system and cardiovascular reactivity. In this case, the postoperative period was uneventful. 

## Conclusions

This case illustrates a successful cesarean delivery in a patient with hEDS under general anesthesia.

A multidisciplinary approach and timely decision-making during the peripartum period ensure safe management in the hEDS setting. There are no general recommendations regarding the type of delivery and anesthesia for patients with hEDS. Temporomandibular joint luxation, cervical spine instability, risk of pneumothorax, and oral/laryngeal hematomas are possible complications of general anesthesia. Spinal pathology may condition the technique for neuraxial anesthesia and there is an increased risk of PDPH and resistance to local anesthetic.
